# Microbial changes linked to the accelerated degradation of the herbicide atrazine in a range of temperate soils

**DOI:** 10.1007/s11356-017-8377-y

**Published:** 2017-01-20

**Authors:** R. L. Yale, M. Sapp, C. J. Sinclair, J. W. B. Moir

**Affiliations:** 1 CRD, Mallard House, 3 Peasholme Green, York, YO1 7PX UK; 2 Department of Biology, University of York, Heslington, York, YO10 5DD UK; 3 FERA Science Ltd., Sand Hutton, York, YO41 1LZ UK; 4 Heinrich-Heine-Universität Düsseldorf, Universitätsstrasse 1, 40225 Düsseldorf, NRW Germany

**Keywords:** Microbial communities, Atrazine, Risk assessment, Fate modelling, Soil pH, Adaptation

## Abstract

Accelerated degradation is the increased breakdown of a pesticide upon its repeated application, which has consequences for the environmental fate of pesticides. The herbicide atrazine was repeatedly applied to soils previously untreated with *s*-triazines for >5 years. A single application of atrazine, at an agriculturally relevant concentration, was sufficient to induce its rapid dissipation. Soils, with a range of physico-chemical properties and agricultural histories, showed similar degradation kinetics, with the half-life of atrazine decreasing from an average of 25 days after the first application to <2 days after the second. A mathematical model was developed to fit the atrazine-degrading kinetics, which incorporated the exponential growth of atrazine-degrading organisms. Despite the similar rates of degradation, the repertoire of atrazine-degrading genes varied between soils. Only a small portion of the bacterial community had the capacity for atrazine degradation. Overall, the microbial community was not significantly affected by atrazine treatment. One soil, characterised by low pH, did not exhibit accelerated degradation, and atrazine-degrading genes were not detected. Neutralisation of this soil restored accelerated degradation and the atrazine-degrading genes became detectable. This illustrates the potential for accelerated degradation to manifest when conditions become favourable. Additionally, the occurrence of accelerated degradation under agriculturally relevant concentrations supports the consideration of the phenomena in environmental risk assessments.

**Electronic supplementary material:**

The online version of this article (doi:10.1007/s11356-017-8377-y) contains supplementary material, which is available to authorized users.

## Introduction

In the EU registration, a new plant protection product requires a risk assessment which includes evaluation of its environmental fate (EC 2009). This is strongly affected by degradation processes (Katayama et al. 2010) which are often mediated by microorganisms (Dodge et al. 2012; Howell et al. 2014). Upon the repeated application of a pesticide or its analogue, microbial communities are able to adapt and degrade the pesticide a faster rate, referred to as accelerated degradation (Racke 1990). Accelerated degradation has been shown to occur for a broad range of pesticides classes (Arbeli and Fuentes 2007), although it is not currently considered in the EU registration studies. For example, to determine the transformation of a chemical in soil, such studies specify that the soils used for the test must not been treated with the substance or its analogues for 4 years (OECD 2002); however, it has been shown that the capacity for pesticide degradation can be maintained for up to 10 years (Cheyns et al. 2012).

Accelerated degradation has the potential not only to reduce pesticide persistence in the environment (Shaner et al. 2007) but also to deplete the efficacy of a pesticide (Krutz et al. 2008). This may contribute to pressure on pesticide use in the light of the limited number of active substances (Chapman 2014) and increased pest resistance (Heap 2016). The phenomenon is dependent on the microbial community; however, the specific changes that occur in the microbial community between pesticide applications and lead to the faster rate of degradation are poorly understood (Arbeli and Fuentes 2007; Itoh et al. 2014).

The herbicide atrazine (1-chloro-3-etylamino-5-isohpropylamino-2, 4, 6-triazine) was selected as a model pesticide to determine the changes that occurred in the microbial community during accelerated degradation. Atrazine was selected as it is highly effective and extensively used around the world (Syngenta 2016), although banned in the EU since 2003 (EC 2015). Additionally, its microbial-degrading pathway is well characterised (Udikovic-Kolic et al. 2012), see Online Resource 1, which enabled the microbial capacity for the degradation of atrazine to be tracked.

Previous studies that have examined the accelerated degradation of atrazine used high concentrations of atrazine to isolate highly tolerant microorganisms for bioremediation (Cai et al. 2003; Wang et al. 2014), often around agrochemical factories (Udikovic-Kolic et al. 2008, 2010) or atrazine-treated agricultural fields (Zablotowicz et al. 2007). This may have biased microbial changes in favour of the fastest growing and most readily culturable members of the community (Dunbar et al. 1997). We used soils with no documented history of *s*-triazine application and applied atrazine at an agriculturally relevant rate to gain an insight into the potential for accelerated degradation in soils that have previously been un-treated or at least have not recently had concerted exposure to atrazine. We then tracked the microbial changes linked to accelerated degradation as it manifested. Pyrosequencing of the 16S rRNA bacterial gene was adopted to enable the community to be analysed more comprehensively compared to fingerprinting techniques such as fatty acid methyl ester (FAME) which had been done previously (Zablotowicz et al. 2007) and which have been shown to lack resolution (Bent et al. 2007). We concentrated on the bacterial portion of the community, as they have been shown to be mainly responsible for accelerated degradation (Walker 1993).

A broad range of soil properties have been associated with influencing accelerated degradation from plant exudates (Piutti et al. 2002) to moisture content (Schroll et al. 2006) and carbon availability in the soil (Popov et al. 2005; Ngigi et al. 2013). Specifically for atrazine, soil pH has shown to affect degradation (Houot et al. 2000). The effect of pH has been examined previously, but not linked to detection of the atrazine-degrading genes or the manifestation of accelerated degradation.

Currently, there is no attempt to account for accelerated degradation in the models used for pesticide dissipation for regulatory purposes, despite the potentially enormous influence of adaptive, biological pesticide degradation on both product effectiveness and residual concentration in soils. To address this, we developed a growth-linked model based on the accumulation of pesticide-degrading microorganisms to fit the kinetics of atrazine disappearance and to facilitate incorporation of accelerated degradation into environmental risk assessments.

## Materials and methods

Three major groups of experiments were conducted in this study: the first to examine the microbial changes associated with accelerated degradation, the second to determine the effect of soil properties on accelerated degradation and the third to explore the effect of pH on accelerated degradation. All three groups of experiments monitored atrazine dissipation to determine the capacity of the soils for accelerated degradation and the presence of the atrazine-degrading genes to track accelerated degradation. The soils used for each experiment are detailed in Table 1, and the sampling regime is detailed in Online Resource 2.

**Table 1:** Identification and properties of the soils used in this study

Soil no.	Farm	Year collected	Soil history	Textural class (USDA)	Sand (%)	Silt (%)	Clay (%)	Total N (%)	OC (%)	C/N ratio	pH (In H_2_O)	Moisture content (g water)^c^
GA_2012^a^	Ganthorpe	2012	A	SL	79	12	9	0.14	1.4	10	6.39	0.12
GS_2012^a^			S	SL	81	8	11	0.14	1.4	10	6.26	0.07
GA	Ganthorpe	2013	A	SL	79	10	11	0.11	1.2	10.9	6.55	0.15
CS	Cotril		S	SL	77	12	11	0.19	1.9	10	6.32	0.22
CA	Cotril		A	LS	83	8	9	0.12	1.3	10.8	6.64	0.16
MS	Mount		S	SCL	54	24	22	0.23	2.2	9.6	8.14	0.22
MA	Mount		A	SCL	51	24	25	0.24	2.8	11.7	7.43	0.25
GRS	Grange		S	SL	65	20	15	0.21	2.7	12.9	5.43	0.29
GRA	Grange		A	SL	67	22	11	0.14	1.9	13.6	6.53	0.20
GRA_pH^b^	Grange	2014	A	SL	62	24	14	0.16	1.4	8.8	6.23	0.18
GRS_pH^b^	Grange		S	SCL	55	26	19	0.33	3.7	11.2	5.39	0.23

Atrazine degradation and the presence of the atrazine-degrading genes was monitored in all soils

*S* set-aside soil, *A* agricultural soil

^a^Soils used to monitor microbial community changes (ATP, Q-PCR and Pyrosequencing)

^b^Soils used to monitor the effect of pH on accelerated degradation

^c^Per gramme of dry soil (105 °C)

### Soil collection

Nine soils which had no documented history of being treated with *s*-triazines were collected from four UK farms, with different physical and chemical properties; Cotril (C): 54° 8′ 2.832″ N, 0° 58′ 36.098″ W; Mount (M): 54° 5′ 36.218″ N, 1° 1′ 38.770″ W; Grange (GR): 54° 6′ 10.703″ N, 0° 50′ 9.082″ W; and Ganthorpe (G): 54° 7′ 27.026″ N, 0° 56′ 48.793″ W. The latitude and longitude for each farm were determined from postcodes inputted into http://www.latlong.net/ (LatLong 2012–2014) and converted into coordinates using http://www.sunearthtools.com (SunEarthTools.com 2009–2016).

From each farm, two soils with different management histories were removed. One soil had been out of agricultural practice for over 5 years and was referred to as set aside (S) while the other soil, which had been under continuous agricultural practice, including pesticide treatment for over 5 years (Online Resource 3), was referred to as the agricultural (A) soil. The set-aside soils had slightly different management histories as follows: Cotril set aside (CS) and Grange set aside (GRS) were grassland, while Mount set aside (MS) was a buffer strip and Ganthorpe set aside (GS) was fallow.

All soils were collected in 2013 apart from the Ganthorpe soils, which were first collected in 2012, and soil from the agricultural site was re-sampled in 2013 to see if the repertoire of pesticide-degrading genes had changed. In addition, the soil for the pH study was collected from Grange in 2014 (Table 1). At each field site, the debris was removed from the soil surface and then ∼10 kg of soil from the top 10 cm was transferred into bags and kept at 4 °C for up to 12 h prior to processing.

### Soil characterisation

Soil pH was measured in H_2_O in 1:2.5 *w*/*v* suspensions (Avery and Bascomb 1974) using a pH probe. The pH probe was calibrated and 10 g of soil added into a 50-mL polyethene beaker with 25 mL of distilled water, stirred and left to stand for 10 min. The pH probe was then introduced and recorded when stable. The moisture content of each soil upon sampling was determined in grammes water per gramme of oven-dried soil (105 °C), shown in Table 1, while the maximum water holding capacity was determined using the Avery and Bascomb method (Avery and Bascomb 1974).

Total organic carbon was measured using the Walkley and Black method (Walkley and Black 1934), total nitrogen using the AOAC method (AOAC 1990) and the soil textural class USDA using the Black method (Black 1965), conducted by Natural Resource Management (NRM) Ltd., Berkshire, UK.

### Microcosm construction

Soils were processed in accordance with OECD 307 guidelines for analysis of chemical transformation in soils (OECD 2002) as follows: soils were air-dried then sieved to 2 mm, moisture adjusted to 40–60% of the MWHC and were maintained at 20 °C ± 2 °C in the dark. The soils Ganthorpe agricultural collected in 2012 (GA_2012) and GS collected in 2012 (GS_2012) consisted of 12 treated sub-samples and 4 control samples—the extra treated samples enabled an assessment of the variation in atrazine recoveries between replicates to be made. For the soils collected in 2013 and 2014, eight sub-samples of 400 g (on a dry weight basis) were transferred into glass amber jars secured with foam bungs. For each soil, four jars were treated with atrazine and four jars were untreated controls.

As a sterile control, 4 × 10 g of Grange agricultural (GRA) soil replicates was autoclaved at 121 °C for 15 min, treated with atrazine as above and sampled in quadruplicate at 0, 1, 3, 7 and 14 days.

### pH adjustment

The soils GRA_pH and GRS_pH had their pH altered according to the method applied by Nicol et al. (2008). GRS (pH 5.4) had 2 mg g^−1^ of lime (Ca(OH)_2_) added to maintain ∼pH 7 (Online Resource 4) and was referred to as GRS amended (GRSa). The GRA_pH soil (pH 6.2) had 8 mg g^−1^ of aluminium sulphate (Al_2_(SO_4_)_3_) added and was maintained at ∼ pH 4 throughout the study (Online Resource 4) and was referred to as GRA amended (GRAa). Soil pH was monitored in a non-atrazine-treated control pot weekly for each soil and each amendment added as required, followed by moisture adjustments.

### Atrazine application

Atrazine (PESTANAL, Sigma-Aldrich) was applied to 4 amber jars per soil type (12 for the GA_2012 and GS_2012). Due to its low water solubility, atrazine was dissolved in methanol and added to 5 g of 1 mm silica sand. The methanol was left to evaporate and the sand mixed into the soil samples. Atrazine was applied at a final agriculturally relevant concentration of 6 μg g^−1^ of dry soil (Tomlin 2009). The four control samples per soil had silica sand with evaporated methanol added. Atrazine was applied in this way three times (twice for the pH study) over an interval of 60 days for the first application and 28 days between the second and third application.

### Atrazine extraction and detection

Samples were removed for analysis 0, 1, 3, 7, 14, 28, 45 and 60 days (day 45 samples were not removed for the GA_2012 and GS_2012) after the first atrazine application and 0, 1, 3, 7, 14, 28 days after the second and third application.

Atrazine was extracted from 1 g (dry weight basis) soil sub-samples by homogenisation with 20 mL of methanol and shaken on a side-side shaker at 230 rpm for 30 min. Following centrifugation (2500 rpm for 5 min), 10 mL of the supernatant was filtered (cellulose acetate 0.45 μm) and 2 mL of the filtrate concentrated to dryness under a flow of nitrogen at 35 °C. The residue was then re-suspended in 200 μL of methanol/water (50:50) using a vortex mixer. Extracts were transferred to HPLC vials and stored at −20 °C prior to analysis.

The concentration of atrazine was determined on the Agilent 1100 series and 1200 series UV module HPLC using a methanol/water mobile phase (50:50) at 1 mL min^−1^ and injection volume of 20 μL, separated on a C18 column with UV detection of atrazine at 222 nm after ∼8.3 min. The estimated limit of detection (LOD) based on the lowest calibration standard was 0.02 μg mL^−1^. The chromatograms were manually integrated using the Chemstation software in order to estimate peak areas, which were then converted into concentrations from calibration curves. Calibration curves were prepared by producing atrazine standards in methanol/water (50:50) at six concentrations from 0.02 to 5.0 μg mL^−1^. Plots of atrazine concentration vs. peak area were constructed, and linear regression was used for determining the atrazine concentration in the samples.

### Modelling of atrazine dissipation

Modelling of atrazine dissipation over the three applications and in the pH and sterile control samples was conducted according to the recommendations of the forum for the co-ordination of pesticide fate models and their use (FOCUS 2006) to obtain estimated values of the dissipation time 50 (DT_50_). The percentage recoveries of atrazine from the theoretical amount applied were modelled using the KinGUii software v2. Initially, the data was optimised to fit the single first-order (SFO) model. The visual fit of the data, *χ* ^2^ value and spread of the residuals were used to determine if a biphasic model would be a better suited to the data, dependent on whether 10% of the initial measured concentration had been reached (FOCUS 2006). A biphasic model was only fitted to the data if compared to SFO the biphasic model resulted in an improved visual fit, low chi-squared (*χ* ^2^) estimate (<15%) and the model parameters passed the *t* test (FOCUS 2006).

To take into account the impact of accelerated degradation on atrazine removal, an alternative approach to modelling the atrazine degradation data was developed, referred to as the ‘growth-linked model’. This involved accounting for the increase in a community of biological atrazine degraders over time during the incubation of soils, as accelerated degradation has been associated with an increase in degrader abundance (Bending et al. 2001). The model consisted of two rates of degradation: (i) a first-order exponential decay rate (chemical) and (ii) a biological decay rate, dependent hyperbolically on atrazine concentration. The difference in atrazine concentration [Atr] between two time points (time *t*, and time *t* + *n*) was calculated computationally as follows

$$ \left[Atr\right]\left(t+n\right)\kern0.5em =\left[Atr\right]t-\left(Y\times n\times {k}_1\times {e}^{-k1t}\right)-\left(N\times n\times V\times \left[Atr\right]\div \left(\left[Atr\right]+{K}_s\right)\right) $$

For the exponential term, *Y* is the percentage of atrazine that is available for degradation, *n* is the length of the time step (typically set at 0.01 days) used in the modelling and *k*_1_ is the exponential atrazine decay rate. For the hyperbolic term, *N* is the size of the atrazine degrader community, *V* is the maximum rate of atrazine removal and *K*_*s*_ is the Michaelis constant representing the concentration of atrazine that gives half the maximum rate of hyperbolic atrazine degradation. The size of the atrazine degrader community (*N*) changes over time as the community of atrazine-degrading organisms grows (as the soil community adapts to atrazine being available). *N* is calculated as a number between 0 and 100 by the following equation

$$ N\kern0.5em ={N_0}^{e\mu t} $$

where *μ* is the exponential growth rate of atrazine degraders, and *N*_0_ is the initial size of the atrazine-degrading community capable of growth. *N* is limited to a maximum arbitrary size of 100.

The model was implemented using a custom-made script written in Python, and the parameters were determined based on qualitative fit to the data.

### Sorption

To investigate the effect of soil pH on sorption of atrazine, batch sorption experiments were conducted as described in OECD 106 (OECD 2000). Prior to the batch sorption experiment, atrazine was determined to be stable in 0.01 M CaCl_2_ for at least 24 h. Sorption of the atrazine was estimated for the GRS_pH and GRA_pH soils and following alteration of their pH GRSa and GRAa, using the standard batch sorption method detailed in OECD 106 (OECD 2000).

Five grammes of each soil, in duplicate tubes, were pre-equilibrated with 22.5 mL of 0.01 M CaCl_2_ by shaking at 200 rpm on a side to side shaker overnight. Atrazine stock solutions of 0.2, 5.0 and 20.0 μg mL^−1^ were added in either 2.5 or 1 mL volumes to obtain theoretical concentrations of 0.02, 0.08, 0.20, 0.80 and 2.0 μg mL^−1^. Additional 0.01 M CaCl_2_ was added to ensure that all tubes had 25 mL of 0.01 M CaCl_2_ to achieve a 1:5 soil to solution ratio. After atrazine addition soil suspensions were returned to the shaker for 24 h to reach pseudo-equilibrium. The samples were then centrifuged at 3000 rpm for 5 min, and the supernatants then filtered (0.2-μm PTFE membrane filters) into 2 mL HPLC vials which were stored at 4 °C prior to analysis.

The final solute concentration of atrazine in solution after adsorption (*C*_aq_) was determined from the HPLC of the supernatant, assuming that all atrazine removed from solution, has been adsorbed. The concentration sorbed to soil (*C*_s_) was calculated as follows

$$ {C}_{\mathrm{s}}\kern0.5em ={K}_{\mathrm{f}}\times {C_{\mathrm{e}}}^{1/n} $$

Values for the Freundlich adsorption coefficient (*K*_f_) and the regression constant (*n*) for the Freundlich adsorption equation were obtained using a solver in Excel by selecting values that minimised the sum of the least squares between measured and modelled values. *C*_aq_ values were then plotted against *C*_s_ to examine the change in sorption as a function of concentration.

### Measuring ATP

Total adenosine triphosphate (ATP) was extracted from 7 days after each application in triplicate from GA_2012 and GS_2012 to monitor the total microbial activity. The Celsis Beverage Kit™ (Brussels, Belgium) was used to measure ATP and the positive control kit (Celsis) used to check the functioning of the Celsis Cellscan M201B luminometer. For the positive control sample, 1 g of sterilised soil was mixed with 10 mL of nuclease-free water (Severn biotech Ltd., Worcestershire, UK) and 100 μL of *Escherichia coli* (NCTC 9703) cell suspension, while a blank cuvette was used as a negative control. The ATP content of 1 g (dry weight) of soil was used for all reactions, 10 mL of nuclease-free water (Severn biotech Ltd., Worcestershire, UK) was added and samples were shaken and processed using the Celsis Beverage Kit. Initially, the variability of ATP (measured in relative light units) was measured in three sub-samples of the same atrazine un-treated soil sample, two aliquots of the soil sludge were then recorded and the variation between the sub-samples was determined not to be significant, using a Student’s *t* test; all *p* > 0.20 (Online Resource 5). One gramme sub-samples from atrazine-treated pots were monitored in triplicate 7 days after each application.

### DNA extraction and PCR

Total community DNA was isolated from the atrazine-treated and control soils across the three applications of atrazine (two applications for the pH study). Approximately 5 g of soil per sample was homogenised in an automatic shaker (Merris Engineering Ltd., Galway, UK) for 2 min, with 10 mL of cetyltrimethylammonium bromide (CTAB) buffer (120 mM sodium phosphate buffer pH 8, 2% CTAB, 1.5 M NaCl), 0.3 mL of antifoam B (Sigma-Aldrich, Dorset, UK) and 10 metal ball bearings (10 mm diameter). The supernatant was removed and centrifuged at 2000×*g* for 2 min and vortexed with 250 μL of Food Buffer B (Promega, Madison, USA) until it appeared milky. This was followed by addition of 750 μL of Precipitation Buffer (Promega) which was vortexed and centrifuged at 13,000×*g* for 10 min. The extracted DNA was then purified using the Promega wizard food kit, in conjunction with the Kingfisher™ mL system (Thermo Fisher Scientific Inc., MA, USA) with a magnetic particle processor using the ‘gDNAnew’ programme. The programme was as follows: 750 μL of the cleared sample was mixed with 600 μL of isopropanol with 50 μL of the magnesil beads (Promega) for 10 min and the genomic DNA bound to the magnetic particles, transferred to 1 mL of lysis buffer B (Promega) for 2 min, followed by four washes in 1 mL of 70% ethanol for 2 min each, followed by 5 min of heating at 65 °C and final elution in 200 μl of TE buffer (pH 8). The purity of extracted DNA was determined using the nanodrop (ND 1000 3.3) system (Thermo Fisher Scientific Inc.) and frozen at −20 °C in TE buffer (10 mM Tris, 1 mM EDTA, pH 8).

### Detection and sequencing of atrazine-degrading genes

Samples from different time points across the three applications of atrazine (two applications for the pH study) were checked for atrazine-degrading genes (Online Resource 1). The polymerase chain reaction (PCR) mix consisted of 1× KAPA HiFi fidelity buffer (Kapa Biosystems, Woburn, MA, USA), 0.3 μM of dNTPs, 0.3 μM of each primer (Table 2), 1 U μL^−1^ KAPA HiFi polymerase and nuclease-free water (Severn Biotech Ltd., Worcestershire, UK) to reach 25 μL final volume. The PCR followed these thermal cycling conditions: initial denaturation at 95 °C for 5 min and 30 cycles of denaturation at 98 °C for 30 s, annealing for 15 s (at the specified temperature in Table 2) and 15 s elongation at 72 °C, followed by a final extension of 5 min at 72 °C (Bio-Rad Laboratories, Inc., USA). Products were visualised on a 2% agarose gel, containing 0.5 μg mL^−1^ ethidium bromide for DNA binding. Bands of the expected size were gel extracted using the Qiagen Gel Purification Kit (Qiagen, Hilden, Germany) and quantified using nanodrop v3.3. Amplicons at concentrations of 4–10 ng μl^−1^ were re-suspended in nuclease-free water (Severn Biotech Ltd., Worcestershire, UK) and 0.3 μM of the forward primer added and directly sequenced using the Applied Biosystems Instrument 3130XL (CA, USA). DNA sequences were analysed using the Sequence Scanner 1.0 software, and similarity to previously sequenced genes was determined from the NCBI using the nucleotide BLAST tool (Altschul et al. 1990).

**Table 2:** Primers used for amplification of the atrazine-degrading genes in PCR and Q-PCR

Gene	Amplicon Length (bp)	Primer name	Primer sequence	Annealing Temp. (°C)	Reference
atzA	500	atzA_F	CCATGTGAACCAGATCCT	55.7	De Souza et al. (1998)
		atzA_R	TGAAGCGTCCACATTACC		
trzN	400	Trz_Nf, C190-10	CACCAGCACCTGTACGAAGG	59	Mulbry et al. (2002)
		Trz_Nr, C190-11	GATTCGAACCATTCCAAACG		
atzB	500	atzB_F	TCACCGGGGATGTCGCGGGC	62.4	De Souza et al. (1998)
		atzB_R	CTCTCCCGCATGGCATCGGG		
atzC	600	atzC_F	GCTCACATGCAGGTACTCCA	62.4	De Souza et al. (1998)
		atzC_R	GTACCATATCACCGTTTGCCA		
atzD	202	atzD_F	TCCCACCTGACATCACAAAC	62.4	Devers et al. (2004)
		atzD_R	GGGTCTCGAGGTTTGATTG		
trzD	663	TrzD_F	CACTGCACCATCTTCACC	55	Fruchey et al. (2003)
		TrzD_R	GTTACGAAC CTCACCGTC		
atzE	203	atzE_F	GAGCCTCTGTCCGTAGATCG	60	Devers et al. (2004)
		atzE_R	GATGGCGTGTACCGTTTACC		
atzF	233	atzF_F	ACCAGCCCTTGAATCATCAG	57	Devers et al. (2004)
		atzF_R	TATTGTCCCGATACCCAACG		
16_Q rRNA	161	16S_qPCR_F	TGGAGCATGTGGTTTAATTCGA	–	Yang et al. (2002)
		16S_qPCR_R	TGCGGGACTTAACCCAACA		
TrzN_Q	70	TrzN_Q_F	GCTTCTGCGACGACCTGTTC	–	In this study
		TrzN_Q_R	TGGTCGATGAGACCCAG		

The hydrolytic enzymes that are encoded by each gene are: AtzA/TrzN, atrazine chlorohydrolase; AtzB, hydroxyatrazine hydrolase; AtzC, *N*-isopropylammelide hydrolase; AtzD/TrzD, cyanuric acid hydrolase; AtzE, biuret hydrolase; and AtzF, allophanate hydrolase

Genomic DNA that did not produce a detectable atrazine-degrading gene product was tested for the effect of inhibitors by adding 2 μL aliquots of the potentially ‘inhibitory’ genomic DNA to a working PCR using undiluted and diluted DNA extracts (1:10 and 1:100); failure to produce a PCR product in an initially working PCR following addition of the inhibitory genomic DNA would indicate that the gene may not be absent in that gDNA sample, but its amplification may have been prevented by inhibition. However, in this study, no inhibition of the PCR was evident.

### Real-time PCR

Relative quantification of *trzN* was performed by Q-PCR to estimate the proportion of the community in the soils GA_2012 and GS_2012 containing the atrazine-degrading gene. The *trzN* gene was selected as the gene of interest; it was the most commonly identified atrazine-degrading gene in this study, in-line with previous studies (Arbeli and Fuentes 2010).

The 16S ribosomal RNA gene was selected as a normalisation gene, due to its presence in all bacteria, although different copy numbers are found in some species (Acinas et al. 2004). Quantification of *trzN* could then be compared between different samples, despite differences in the number of bacteria and or concentration of gDNA template. Primers for the gene targets (TrzN_Q_F & R,. 16S_Q_F & R, the latter from Yang et al. 2002) were selected using the Primer Express® Software for Real-Time PCR version 3.0 (Applied Biosystems) and synthesised by Eurofins MWG Operon. Primers are listed in Table 2. The Q-PCR method was adapted from Udikovic-Kolic et al. (2010) and was performed on an Applied Biosystems StepOne™ instrument using SYBR Green® for detection in 20 μL reactions. Each reaction consisted of 10 μL of Power SYBR® Green Mix 2× (Applied Biosystems), 6.2 μL nuclease-free dH_2_O (Ambion®), 0.4 μL of each primer (5 μM each) and 3 μL of gDNA. Reactions were run in 96-Well Optical Reaction Plates (Applied Biosystems) for relative quantification, according to the manufacturer’s instructions.

Thermal cycling conditions were as follows: hold at 95 °C for 10 min, 40 cycles at 95 °C for 15 s and 60 °C for 1 min. The final step was added initially to produce a melt curve, starting from 60 °C to 95 °C to ensure that a single product was produced. Each sample was run in triplicate per target to obtain average Ct (cycle threshold) values.

Standard curves of *trzN* and *16S rRNA* were constructed using purified PCR products for these genes amplified from a soil sample that had been treated with atrazine three times. The PCR amplicons were purified using the Qiagen gel purification kit and quantified based on absorbance at 260 nm using Nanodrop v3.3. Amplicons were required to have a 260/280 ratio between 1.8 and 2.0. For relative quantification standard curves were produced by serially diluting the amplicons; tenfold-four times for *trzN* and tenfold-five times for *16S rRNA*. The standard curves *16S rRNA* and *trzN* are shown in Online Resources 6 and 7, respectively. Plots of log DNA concentration vs. Ct value were constructed and the linear regression line used for determining the gene concentration in the sample. The percentage of the bacterial community containing *trzN* was then calculated using the following formula

$$ \frac{trzN\mathrm{gene}\kern0.5em \mathrm{concentration}}{16S rRNA\ \mathrm{gene}\ \mathrm{concentration}}\times \frac{\mathrm{length}\ \mathrm{of}\kern0.5em 16\mathrm{S}\mathrm{gene}\mathrm{product}\left(\mathrm{bp}\right)}{\mathrm{length}\ \mathrm{of}\  trzN\mathrm{gene}\ \mathrm{product}\left(\mathrm{bp}\right)}\times 100 $$

Significant differences in the portion of the community containing *trzN* between treated and control samples, for each soil, were determined by unpaired *t* tests.

### Community analysis

To determine the bacteria present in the GA_2012 and GS_2012 soils, the V3-V5 fragment of the 16S rRNA gene was amplified by the PCR and pyrosequenced using primers previously tested by Klindworth et al. (2013). PCR primers were adapted to 454 amplicon sequencing, for which a M13 adapter (bold and underlined) was attached to the target forward primer Bakt_341F (5′-**CACGACGTTGTAAAACGAC**CCTACGGGNGGCWGCAG-3′). To aid multiplexing different samples, different barcodes were included using the M13 adapter. Sequence adapter A (bold) was followed by the 454 amplicon sequencing specific 4-mer amplification key (italics) followed by a 10-mer sequence (NNNN) barcode and M13 (bold and underlined) (5′-**CCATCTCATCCCTGCGTGTCTCCGAC** *TCAG*NNNN**CACGACGTTGTAAAACGAC**-3′). An overview of barcode sequences used can be found in Online Resource 8. The 25-mer Lib-L specific sequence adapter B was followed by the reverse template specific primer sequence Bakt_805R (italics) (5′-CCTATCCCCTGTGTGCCTTGGCAGTC*GACTACHVGGGTATCTAATCC*-3′).

The PCR mix consisted of 1× KAPA HiFi fidelity buffer (Kapa Biosystems, Woburn, MA, USA), 0.3 μM of dNTPs, 0.3 μM of each primer, 1 U μL^−1^ KAPA HiFi polymerase, 0.3 μM M13 adapter and nuclease-free water (Severn Biotech Ltd., Worcestershire, UK) to reach 25 μL final volume. The PCR followed these thermal cycling conditions: initial denaturation at 95 °C for 5 min and 30 cycles of denaturation at 98 °C for 30 s, annealing for 15 s at 55.3 °C and 15 s elongation at 72 °C, followed by a final extension of 5 min at 72 °C using the Bio-Rad C1000 (Bio-Rad Laboratories, Inc., USA). Products were visualised on a 1% agarose gel, containing 0.5 μg mL^−1^ ethidium bromide for DNA binding. Band intensity of 16S rRNA gene amplicons of the correct size (469 bp) was used to estimate quantity for pooling. Subsequently, pooled amplicons were run on a 3.5% gel to separate out small fragments, which were excised and extracted using the Qiagen Gel purification kit according to the manufacturer’s instructions. This concentrated pooled sample was heated for 5 min at 95 °C and snap cooled on ice for 2 min. This was followed by a second gel electrophoresis on a 2% gel of the concentrated pooled amplicons. The excised band of correct size was extracted using the Qiagen kit. The purity and concentration was determined using Nanodrop (ND-1000 3.3) and DNA fragment pattern assessed using a DNA 1000 Chip on the Agilent Bioanalyser, 2100 series (Agilent Technologies Inc., CA, USA).

The sequences of partial 16S rRNA genes were obtained using a Roche GS-FLX 454 pyrosequencer (Roche, Mannheim, Germany) using picotitre-sequencing plates and sequenced as advised by the manufacturer for amplicon sequencing. Samples were processed through the quantitative insights into microbial ecology (QIIME) pipeline (Caporaso et al. 2010b). Initially, samples were filtered by quality (−M4, maximum number of primer mismatches; −s 30, minimum average quality score allowed in read) and were split by their barcode sequence. The number of sequences presents before and after quality filtering is shown in Online Resource 9. Clustering into operational taxonomic units (OTUs) was then performed using UCLUST at the 97% similarity level, indicative of species level (Edgar 2010). The most abundant sequences were chosen as being representative of a cluster and aligned with the PYNAST method (Caporaso et al. 2010a). The OTU table generated was then rarefied to 2910 sequences per sample to avoid bias. OTUs present in 1 or 2 samples were removed from the rarefied OTU table (L6), and this table was then transformed by square root and a Bray-Curtis resemblance matrix was constructed in PRIMER6 (Primer-E Ltd., Lutton, UK).

The Bray-Curtis matrix was clustered using hierarchical-clustering with group average linkage to produce a dendrogram representing the scaled similarity between samples. Non-metric multidimensional scaling (nMDS) plots were used to provide a visual representation of the similarities between bacterial communities, based on the Bray-Curtis similarity index. On the nMDS plots, the clustering of data points was highlighted by overlaying ellipses based on the clustering. The significance of bacterial community clustering was quantified using analysis of similarity (ANOSIM) which is an analogue to the standard univariate one-way analysis of variance (ANOVA) designed for ecological data. ANOSIM generates an *R* statistic that indicates the separation between groups where an *R* of 1 indicates complete separation and *R* of 0 indicates that there is no separation (Clarke et al. 2006).

To test the variation in the bacterial community explained by each variable, PERMANOVA was calculated using the Adonis function in the R package vegan (Oksanen et al. 2013). A matrix of variables (atrazine, soil and incubation time) vs. samples was constructed that corresponded to the relative abundance of each OTU in each sample. The test statistic and associated *p* value was calculated using 999 random permutations on the basis of Bray-Curtis distances. A *p* value was calculated using the classical *F* distribution approximation. The significance level to reject the null hypothesis was set a priori to 0.05. Results were visualised using R (version 3.2.1) R Core Team, 2015.

### Principal component analysis

To explore the variation between the nine soils, principal component analysis (PCA) was conducted in PRIMER v6 (Clarke et al. 2006), based on different soil properties. A draftsman plot showed that the data points were equally spread; therefore, multivariate normality was assumed. Each variable was normalised (subtraction of the mean and dividing by the standard deviation) to provide comparable, dimensionless scales for a correlation based PCA. The PCA was composed of five principal components, and the eigenvalues, eigenvectors and principal component scores were used to determine the soil properties that best explained the variation between sites.

### Accession numbers

The 16S rRNA amplicons have been deposited in the SRA with the accession number SRP066748 (PRJNA304340).

## Results

### Soils of different physico-chemical properties exhibited a similar pattern of accelerated degradation

**Fig. 1:**
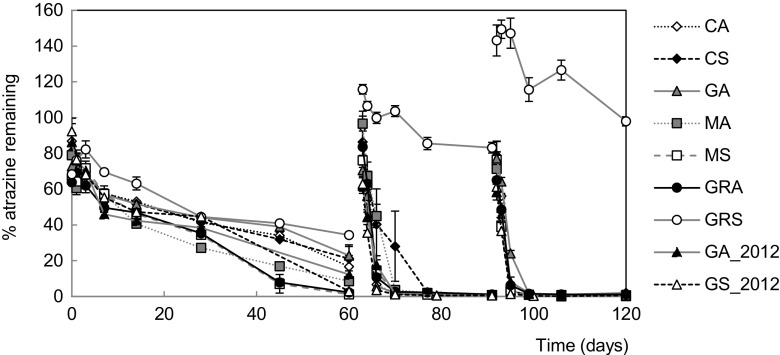
Dissipation of atrazine over three applications to nine temperate soils. Atrazine concentration in soil sub-samples was monitored at regular intervals by HPLC-UV. *Error bars* show the standard error between replicates, *n* = 12 for GA_2012 and GS_2012 applications 1 and 2, *n* = 6 for application 3, and *n* = 4 for all other soils. Soil identifier: *CA* Cotril agricultural, *CS* Cotril set aside, *GA* Ganthorpe agricultural, *MA* Mount agricultural, *MS* Mount set aside, *GRA* Grange agricultural, *GRS* Grange set aside, *GA_2012* Ganthorpe agricultural collected in 2012, *GS_2012* Ganthorpe set aside collected in 2012

Eight out of the nine soils untreated by *s*-triazines for at least 5 years and banned since 2003 (EC 2015) demonstrated accelerated degradation, evident by their faster rate of dissipation following a second application of atrazine (Fig. 1).

Using the European regulatory FOCUS guidance (FOCUS 2006), data from the first application of atrazine fitted single first-order (SFO) kinetics adequately for all soils, with good visual fits and *χ* ^2^ error values of <15% (Table 3). After the second and third applications, several soils had poor SFO visual fits missing several points, and this was not improved by fitting the data to biphasic kinetics. Initially, four soils, CS, Cotril agricultural (CA), GA and GRS, had estimated DT_50_ values of greater than 30 days and the remaining soils had DT_50_ values of greater than 19 days. By the second application, all soils, apart from GRS, had DT_50_ values of 3 days or less (Table 3). After the third atrazine application, all soils, apart from GRS, had DT_50_ values of less than 2 days (Table 3). The parameters used for the SFO fits are in the Online Resource 10.

**Table 3:** Estimated time for 50% of atrazine to degrade in days (DT_50_) and corresponding chi-squared value (*χ* ^2^) for single first-order (SFO) fits for atrazine dissipation in soils that received two or three successive applications of atrazine

Soil	First application		Second application		Third application	
	DT_50_	*χ* ^2^	DT_50_	*χ* ^2^	DT_50_	*χ* ^2^
GS_2012	21.49	10.26	1.76	9.16	1.53	22.24
GA_2012	20.18	11.85	1.08	6. 73	1.01	16.37
CS	32.34	5.72	2.93	12.42	0.98	12.8
CA	30.23	8.2	1.03	10.08	1.3	26.9
MS	19.81	11.09	1.59	17.15	0.91	8.8
MA	19.58	7.84	2.72	5.57	0.97	12.13
GRS	48.14	6.58	57.88	5.66	48.62	5.06
GRA	20.38	10.39	1.39	16.73	1.32	18.61
GA	37.1	10.94	1.13	15.04	1.89	12.26
GRS_pH^a^	46.08	3.81	32.61	9.45	–	–
GRSa^b^	18.25	9.78	1.05	13.94	–	–
GRA_pH^a^	18.04	11.77	1.54	6.64	–	–
GRAa^b^	30.02	10.83	21.29	7.36	–	–

Refer to Table 1 for soil origin

^a^Soils collect from Grange farm in 2014 for the pH experiment

^b^GRSa and GRAa soils originated from GRA_pH and GRS_pH after their pH was amended

All eight soils exhibiting accelerated degradation showed a 7–32-fold reduction in their DT_50_ values from the first to the second application of atrazine, whereas the DT_50_ of GRS was 48.6 days after the third application (Table 3), with the amount of atrazine appearing to accumulate after each application (Fig. 1).

**Fig. 2:**
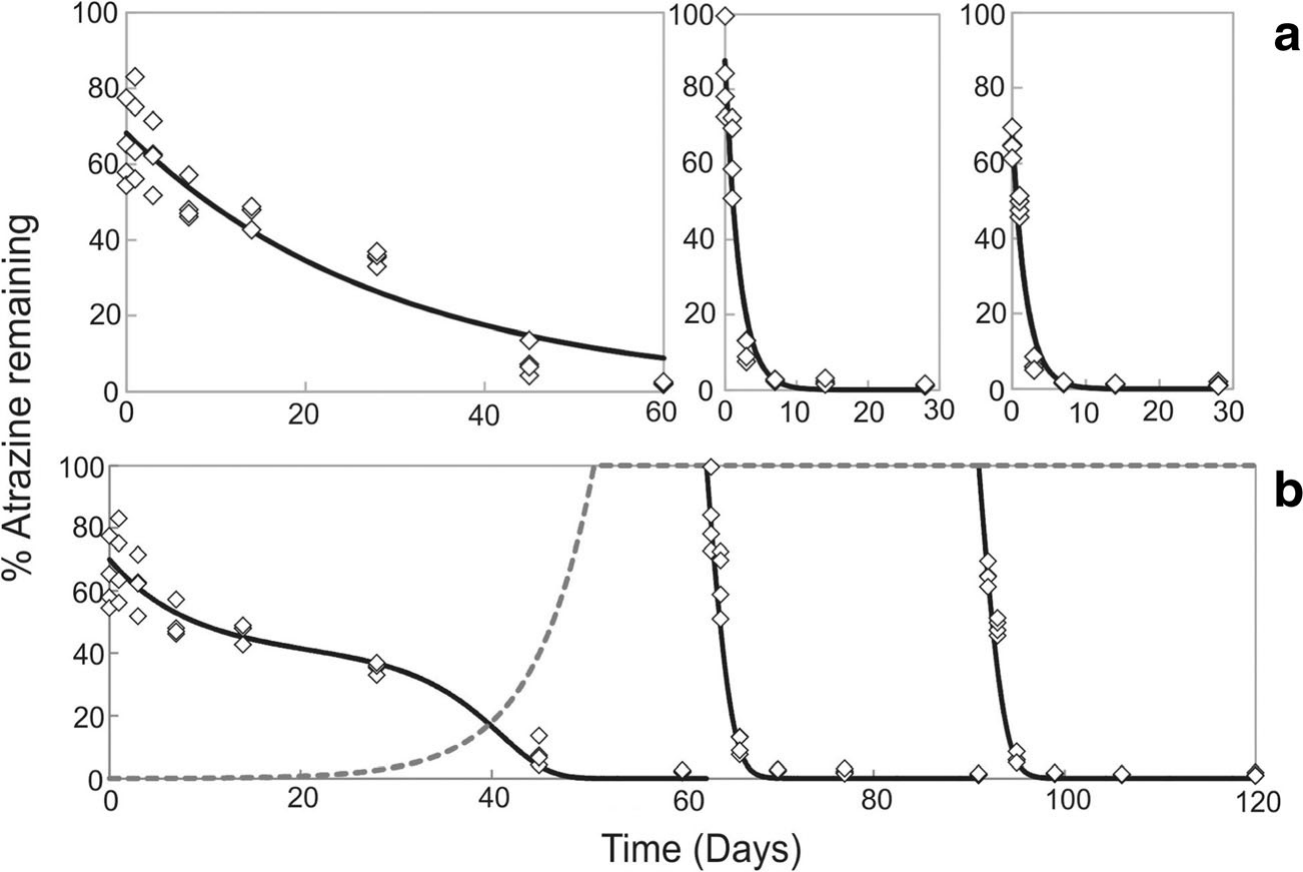
Comparison of modelling approaches for the dissipation of atrazine in GRA over three applications. Using the regulatory single first-order (SFO) approach (**a**), with each application modelled separately and the ‘growth-linked model’ described in this study (**b**). The growth-linked model enabled all applications to be modelled simultaneously. In both modelling approaches, the model fit of % atrazine remaining is shown as a *solid black line* and individual soil sub-samples as *diamonds* (*n* = 4). For the growth-linked model (**b**), the *grey dashed line* represents the number of atrazine degraders

It is evident that the vast majority of these soils are exhibiting an accelerated rate of atrazine degradation within 60 days of applying atrazine. Using a sterile control, it was shown that sterile soil displayed a DT_50_ of 107.7 days compared to 20.4 days in a matched non-sterile soil (Online Resource 11). This confirmed in-line with previous studies that accelerated degradation of atrazine is microbially driven (Zablotowicz et al. 2007). However, the modelling approaches applied above do not take into account the kinetics of microbial growth or microbial adaptation over multiple applications. In fact, the SFO kinetics applied to the first application of atrazine fails to capture the rate of atrazine decay, which clearly begins to accelerate between 28 and 45 or 45 and 60 days for most soils. This is illustrated in the GRA soil in which time points at 28 and 60 days deviate significantly from the exponential fit (Fig. 2). Similar poor fits are also observed during the degradation of the first atrazine application in the other soils (Online Resources 12–18).

Therefore, we addressed this by developing a model that included an initial rate of exponential atrazine degradation and an activity following hyperbolic, Monod-type kinetics, consistent with a microbially mediated biodegradation. This latter activity was dependent on the size of a microbial community, capable of atrazine utilisation. This community was modelled to increase exponentially up to a limit, as would be expected for a microbial community in vivo. This more sophisticated approach was able to capture the change in atrazine degradation for each of the degrading soils and accounted for the variation in rate of degradation between atrazine additions. For example, the GRA data fitted using the microbial growth model fits the data much better than the individual exponential fits for each atrazine application (Fig. 2). The growth-linked model enabled a single set of parameters to be used to fit all the atrazine degradation kinetics, rather than using separate parameters for each atrazine addition.

Overall, the growth-linked model gave a superior fit for the data from all eight soils exhibiting accelerated degradation (Fig. 2 and Online Resources 12–18). The parameter that varied the most widely was the initial number of atrazine degraders in the community (Online Resource 19), which varied by three orders of magnitude (from 0.00005 to 0.03). The other fitted variables had values in the ranges: *k*_1_ from 0.06–0.18, *Y* from 30 to 65%, *μ* from 0.155 to 0.225 day^−1^, *V* from 0.6–0.7 and *K*_s_ = 75 in all cases. The *K*_s_ value of 75 is a percentage of the initial applied atrazine concentration (6 μg L^−1^), i.e. *K*_s_ = 20.9 μM. This result from modelling is remarkable given that the atrazine-degrading enzyme TrzN has been measured to have *K*_m_ = 20 μM (Shapir et al. 2005) and 25 μM (Topp et al. 2000).

### The atrazine-degrading genes were detectable in soils exhibiting accelerated degradation

To determine if the soils exhibiting accelerated degradation carried the atrazine-degrading genes and the extent to which the repertoire of genes varied between soils, DNA from 3, 14 and 28 days after each application of atrazine was tested. All soils that exhibited accelerated degradation contained at least *trzN*, while none of the atrazine-degrading genes were detectable in GRS (Table 4) which did not exhibit accelerated degradation (Fig. 1).

**Table 4:** Summary of the atrazine-degrading genes detected in nine temperate soils, 14 days after the third application of atrazine

Soil	Number of genes detected^a^	atzA (100%)	trzN (100%)	atzB (100%)	atzC (100%)	atzD	trzD (100%)	atzE	atzF
GA_2012	3		+	+			+		
GS_2012	4	+	+	+			+		
CS	3	+	+	+					
CA	3	+	+	+					
MS	1		+						
MA	3	+	+	+					
GRS	0								
GRA	3	+	+	+					
GA	4	+	+	+	+				

Refer to Table 1 for soil origin. Refer to Table 2 for a description of the hydrolytic enzymes that are encoded by each gene

^a^The closest relative for each of the atrazine-degrading genes detected are shown in Online Resource 20

The presence of the atrazine-degrading genes (Online Resource 1) was determined for all nine soils. Soil MS exhibited accelerated degradation (Fig. 1), but only *trzN* could be detected (Table 4) and was characterised by its high clay content of 22% and a low C/N ratio of 9.6 (Table 1).

In five of the soils that exhibited accelerated degradation (GA_2012, Mount agricultural (MA), CA, CS and GRA), three atrazine-degrading genes were detected (Table 4). All these soils had a pH of greater than 6.3 (Table 1). *AtzA* and *atzB* were detected in six of the eight soils showing accelerated degradation (Table 4), while *atzC* was only detected in GA. The gene *trzD* of the lower atrazine-degrading pathway, which is more tightly regulated and less commonly identified (Udikovic-Kolic et al. 2012), was only detected in GA_2012 and GS_2012 (Table 4). It is also notable that re-sampling of the GA_2012 soil in 2013 (GA) led to a different repertoire of atrazine-degrading genes being detectable, with the detection of *atzA* and *atzC* which were below the limit of detection in 2012, while *trzD* was below the limit of detection in 2013. All of the atrazine-degrading genes detected had sequences which were 100% identical to those characterised previously (Online Resource 20).

### Atrazine treatment does not have a gross impact on the overall microbial community

To determine whether the increase in atrazine degradation was due to an overall increase in microbial activity in soils, soil ATP content was measured following repeated applications of atrazine. There was no significant change in ATP concentration in soils over time (Online Resource 21), indicating that a significant increase in the microbial community is unlikely to explain the increased degradation of atrazine and that a more likely reason would be proliferation of specific microorganisms containing the atrazine-degrading genes.

**Fig. 3:**
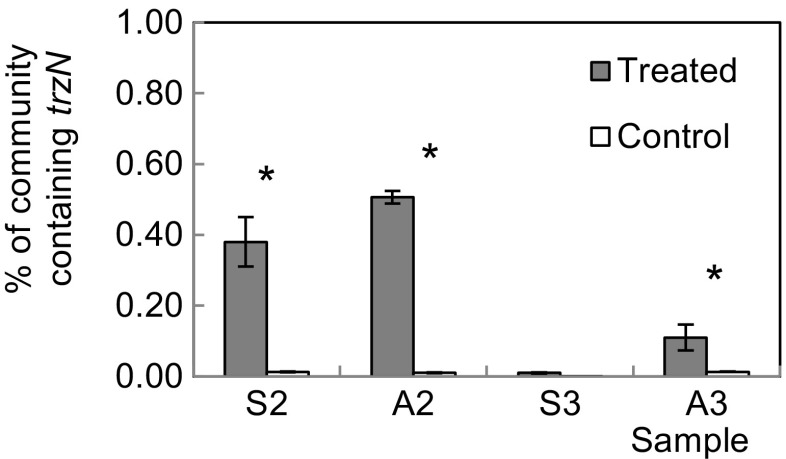
Percentage of the bacterial community that contain the atrazine-degrading gene *trzN* in the GA_2012 and GS_2012 soils. The *trzN* gene was monitored in the Ganthorpe agricultural soil, GA_2012 (*A*), and Ganthorpe set-aside soil, GS_2012 (*S*), 14 days after the second (*2*) or third application (*3*) of atrazine to each soil. *TrzN* was measured in atrazine-treated and control sub-samples. The proportions of the community carrying *trzN* was normalised against the *16S rRNA* gene for each sample. *Error bars* show the standard error between experimental replicates, *n* = 6. The significant differences between the proportion of the community containing *trzN* between treated and control soils are indicated by *asterisk* (*p* < 0.05)

It is clear that there is a significantly greater proportion of the bacterial community containing *trzN* after the second and third application of atrazine in the agricultural soil and after the second application in the set-aside soil (*p* < 0.05) (Fig. 3). It is also evident that only a small proportion (<0.5%) of the bacterial community contained *trzN*.

**Fig. 4:**
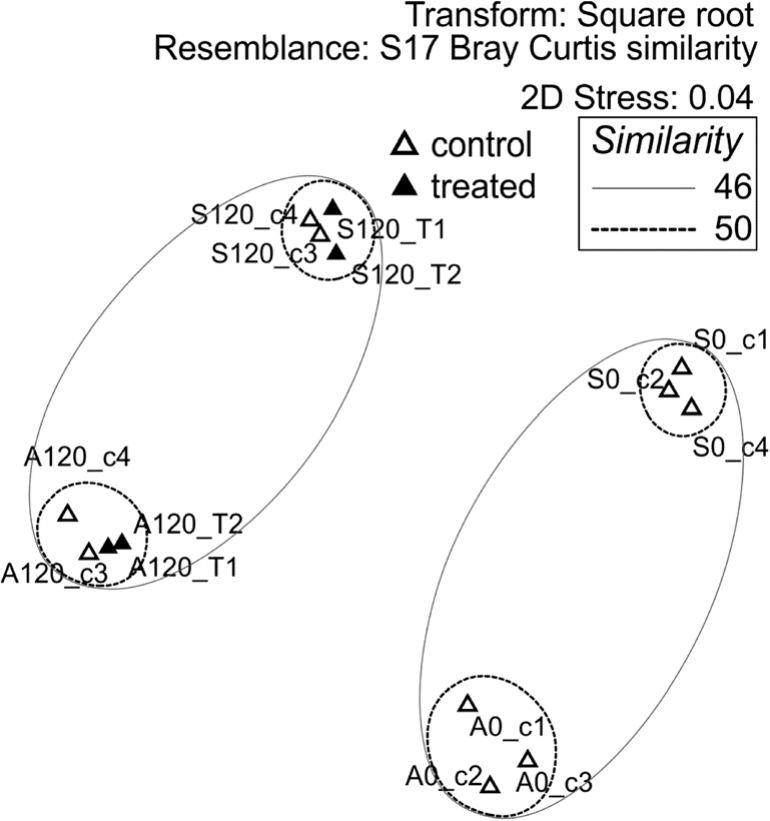
Non-metric multidimensional scaling plot of the association of bacterial communities with atrazine treatment in the GA_2012 and GS_2012 soils. Each bacterial community is represented by a *triangle*, originating from the Ganthorpe agricultural soil, GA_2012 (*A*), and Ganthorpe set-aside soil, GS_2012 (*S*). The bacterial communities are based on OTU clustering of the pyrosequencing of *16S rRNA* genes. The variables included in the analysis were soil history: set aside (*S*) or agricultural (*A*), duration in days under incubation conditions (0 or 120 days) and atrazine treatment, treated (*T*) or control (*C*). The *similarity ellipses* are based on hierarchical clustering shown in the Online Resource 26

Non-metric multidimensional scaling was conducted using ANOSIM to observe clustering of soil samples according to various variables (Fig. 4). The low abundance of *trzN* relative to the overall number of bacteria present is consistent with the absence of significant clustering between samples based on atrazine treatment (ANOSIM; *R* 0.08, *p* 0.25). This is also consistent with analysis by PERMANOVA showing that the variation in the bacterial communities was most affiliated with their duration of incubation (*R* ^2^ = 0.23, *p* 0.0001) and soil type (*R* ^2^ = 0.17, *p* 0.0002) rather than atrazine treatment (*R* ^2^ = 0.08, *p* 0.09).

### GRS was most strongly associated with pH

Atrazine was found to be at least 19 times more persistent in GRS compared to any other soil, following the second and third application of atrazine (Table 3). The soil properties that distinguished this soil from the others that exhibited accelerated degradation were investigated. The nine soils used in this study had been naïve to *s*-triazines for five or more years (Online Resource 3), and the DT_50_ values in the set-aside soils, which had not had pesticides applied, were very similar to those seen in the agricultural soils (Table 3). Therefore, it was unlikely that exposure to pesticides other than *s*-triazines affected accelerated degradation.

PCA was used to determine the soil physical and chemical properties (Table 1) that explained the variation between soils and those most correlated with the GRS soil, which may have affected its ability to mediate accelerated degradation.

**Fig. 5:**
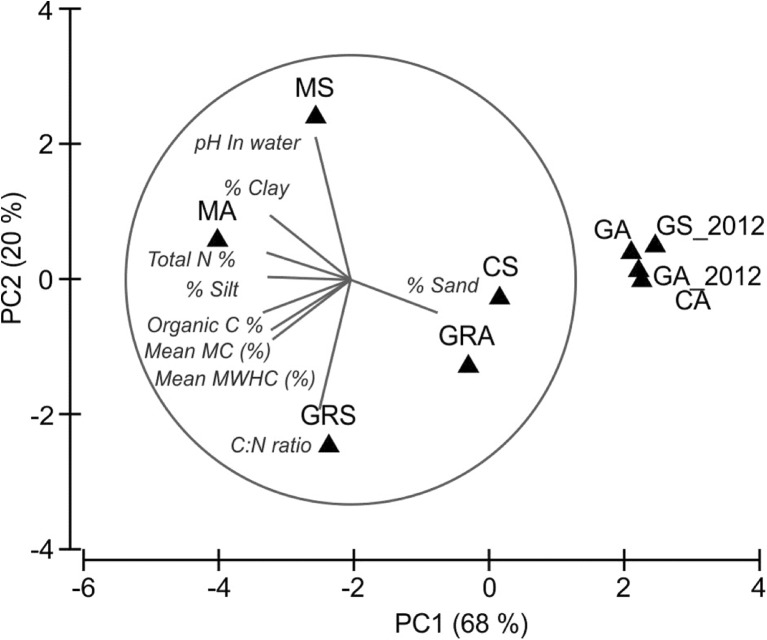
Principal component analysis (PCA) of the association of nine temperate soils with various physico-chemical properties. Measured soil properties were normalised, and the corresponding data matrix was subject to PCA. Each *triangle* represents an individual soil. The association between different soils is plotted along the first two principal components, which represent 68 and 20% of the variation between the soils. Soil properties: *MWHC* maximum water holding capacity, *MC* moisture content, *C/N* ratio carbon/nitrogen ratio, and *total N* total nitrogen. Soil identifiers: *CA* Cotril agricultural, *CS* Cotril set aside, *GA* Ganthorpe agricultural *MA* Mount agricultural, *MS* Mount set aside, *GRA* Grange agricultural, *GRS* Grange set aside, *GA_2012* Ganthorpe agricultural collected in 2012, and *GS_2012* Ganthorpe set aside collected in 2012

It is clear that the seven soils are scattered and do not cluster dependent on the farm they originated from or whether they were collected from set-aside or agricultural sites (Fig. 5). PC1 explained 68% of the variation and PC2, 20%, with most of the remainder explained by PC3 (8.4%), together explaining 96.3% of the variation in soil properties (Online Resource 22). It can be inferred that PC1 is roughly an equal weighted combination of most of the soil properties including texture (sand, silt and clay), nutrients (organic carbon and total nitrogen) and moisture parameters (moisture content (MC) and maximum water holding capacity (MWHC)) with the greatest contribution from sand and organic carbon contents (Online Resource 23).

PC2 is mainly explained by pH, although there is some contribution from carbon to nitrogen ratio (C/N) and moisture content too, while PC3 is mainly explained by C/N ratio (Online Resource 23). Based on the principal component scores, it was clear that the variation between the agricultural soils CA, GA and MA were mainly dependent on PC1 (Online Resource 24). Specifically, CA had the highest sand content, while GA and MA had the lowest and highest organic carbon contents, respectively (Table 1). GRA was an outlier from the other soils mainly affiliated with PC3 (Online Resource 24) and explained by having the highest C/N ratio (Table 1), whereas MS and GRS were strongly affiliated with PC2, due to them having the highest and lowest pH respectively.

### Soil pH affected the capacity of the soils to mediate accelerated degradation of atrazine

The GRS soil was the only one not to exhibit accelerated degradation and was notably the soil with the lowest pH. To determine whether the lack of accelerated degradation was linked to the low pH and whether the genetic potential for atrazine degradation is retained in this low pH soil, the pH of the GR soils was experimentally altered. GRA which had a pH of 6.2 and had exhibited accelerated degradation (Fig. 1) was acidified and maintained at ∼pH 4 to suppress accelerated degradation, while GRS which had a pH of 5.4 and did not exhibit accelerated degradation (Fig. 1) was neutralised and maintained at ∼pH 7 to determine whether accelerated degradation could be induced.

**Fig. 6:**
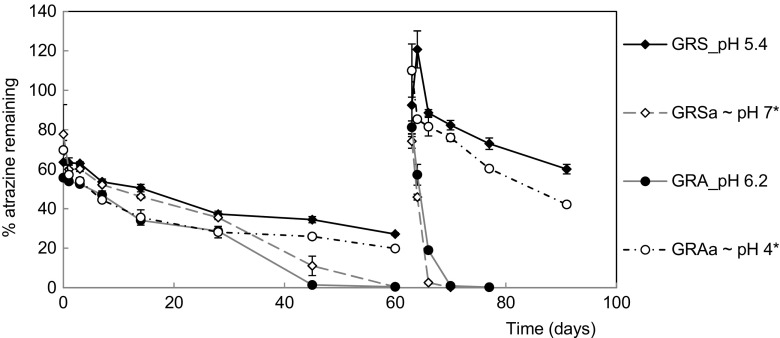
Effect of soil pH on atrazine dissipation over two applications in the GRA_pH and GRS_pH soils. Atrazine was applied to the Grange set-aside soil (GRS_pH) and Grange agricultural soil (GRA_pH) which were collected in 2014 and their pH was amended (-a) to approximately pH 7 (GRSa) and pH 4 (GRAa), respectively. *Error bars* represent the standard error between replicates (*n* = 4). Parameters used for the SFO model fits are provided in the Online Resource 10; *asterisk* shows that the pH of the amended soils (GRSa and GRAa) are only approximate as there was minor variation in their soil pH throughout the experiment (Online Resource 4)

After the second application of atrazine, the concentration of atrazine was below the limit of detection at day 14 and day 28 in GRSa and at day 28 in GRA, showing how rapid accelerated degradation of atrazine had occurred in the amended GRS sample. Accelerated degradation was only observed in GRSa and GRA (Fig. 6) in which both had a pH greater than pH 6.2. The DT_50_ values of these soils were less than 1.6 days after the second application of atrazine although the soils with pH < 5.4. GRS and GRAa exhibited DT_50_ values that were longer than 21 days (Table 3), indicating that atrazine will be more persistent in these soils. In addition to its low pH, GRS is also affiliated with high contents of clay, moisture and organic carbon (Table 1).

Sorption was shown to increase with atrazine concentration (Online Resource 25) and was slightly higher in the acidic soils GRAa and GRS than the neutral soils GRA and GRSa, but these differences were small and insufficient to explain the >7-fold difference in atrazine dissipation rates between these treatments (Table 3 and Fig. 6).

To see if the change in soil pH modified detection of the atrazine-degrading genes in the treated soil PCRs of the atrazine chlorohydrolase genes, *atzA* and *trzN* were conducted. Neither *atzA* nor *trzN* could be detected after multiple applications of atrazine in the acidic soils GRS and GRAa. Contrastingly, both *atzA* and *trzN* were detectable in soils at pH > 6.2 (GRA and GRSa) after two applications of atrazine, indicating that the genes are present in both GRA and GRS but only proliferate to become detectable at near neutral pH.

## Discussion

### Accelerated degradation manifested in soils with no history of *s*-triazine use after one application

Eight soils transitioned to an accelerated rate of dissipation within 60 days (Fig. 1) demonstrating that most soils tested had the capacity for the accelerated degradation of atrazine. The soils had no *s*-triazine history documented, and atrazine has been banned since 2003 (EC 2015). Previous studies have shown that accelerated degradation was evident in soils that had received two annual applications of atrazine in the field, and the rates of mineralisation in these soils were much lower (Zablotowicz et al. 2007; Houot et al. 2000) compared to the very rapid rate of atrazine degradation seen in this study, which could be explained by the shorter interval between applications. In addition, most previous studies that have demonstrated accelerated degradation have used soils with long histories of atrazine applications (Zablotowicz et al. 2007). Although spray drift of simazine, which has been applied more widely in the UK (D. Garthwaite: pesticide usage survey; personal communication), cannot be ruled out as a source of low level *s*-triazine availability stimulating these soils, this does not detract from the finding that accelerated degradation of atrazine occurs in soils that have no recorded history of deliberate *s*-triazine application. Such high potential for the accelerated degradation of atrazine could mean that soils with history of *s*-triazine use over 4 years ago may be ineffective, although this will need to be investigated in a field and the extent to which this applies to other pesticides explored. Additionally, Orlikowska et al. (2015) detected atrazine in marine waters which they presumed to have reached the sea by runoff from agricultural land. An interesting extension to this project would be to investigate the movement of atrazine residues and potentially atrazine-degrading organisms, via artificial drainage or runoff.

### Accelerated degradation was associated with the atrazine-degrading genes

To examine the microbial changes linked to accelerated degradation, general activity, microbial community structure and the capacity for degradation were monitored. No increase in general biological activity was linked to accelerated degradation (Online Resource 21) in agreement with other studies; De Andréa et al. (2013) saw no correlation between dehydrogenase activity and glyphosate mineralisation, while Udikovic-Kolic et al. (2011) demonstrated major shifts in microbial communities treated with atrazine. In this study, we found that there was no significant change in the microbial community in response to atrazine treatment (Fig. 4). This probably relates to the levels of atrazine employed. In the current study, atrazine was used at the recommended agricultural level, whereas the previous analysis concerned a highly contaminated pesticide site (Udikovic-Kolic et al. 2011).

Accelerated degradation was associated with detection of the atrazine-degrading genes of the hydrolytic pathway, which were detected upon the increased rate of dissipation of atrazine. There is an alternative atrazine-degrading pathway, the oxidative-hydrolytic pathway for atrazine dissipation which degrades atrazine via the production of deethylatrazine (DEA) and deisopropylatrazine (DIA) (Giardina et al. 1982). However, the oxidative-hydrolytic pathway has mainly been associated with non-adapted soils that have a slow rate of degradation (Fournier et al. 1997). In addition, hydroxyatrazine has been shown to be the dominant metabolite in atrazine adapted soils (Krutz et al. 2010) and in atrazine-mineralising cultures (Yanze-Kontchou and Gschwind 1994; Mandelbaum et al. 1995; De Souza et al. 1998). PCR was used in an attempt to identify the *thcBCD* genes involved in the oxidative-hydrolytic pathway (Shao and Behki 1996) but were not detected in this study.

### Accelerated degradation of atrazine was associated with a small portion of the microbial community

The repertoire of atrazine-degrading genes was variable between soils and was shown to vary after 1 year between subsequent samples of the GA soil, possibly due to changes in the exact location the soil was removed (Bending et al. 2001). The high degree of variability in apparent gene content between soils (Table 4), yet the similar atrazine degradation rate following adaptation (Fig. 1), suggests that limitations in the method may also have been an issue. This is also reflected in the relatively low percentage of the microbial community composed of atrazine degraders (estimated to be 0.5%), (Fig. 3). However, the low proportion of atrazine degraders may also have been affected by the low concentration of atrazine available for them to degrade. Bælum et al. (2006) saw that microbial growth was more pronounced when the bacterial community was supplied with high pesticide concentrations compared to low concentrations. However, Udikovic-Kolic et al. (2010) found that only 1–4% of their atrazine community contained the atrazine-degrading genes, even when the atrazine concentrations were 100× greater than applied in this study, and Sniegowski et al. (2012) showed that only 0.5% *v*/*v* of a pesticide degradation inoculum was required for maximum pesticide degradation.

### A new microbial growth-linked model enables accelerated degradation to be considered in environmental risk assessments

To enable accelerated degradation to be considered in the risk assessment, we modelled our data in-line with the current regulatory approach for modelling pesticide dissipation to a microbial-based approach. Fitting the disappearance of atrazine, using the standard FOCUS Guidance (FOCUS 2006) used in EU pesticide regulation, showed a drastic reduction in DT_50_ between the first and second application of atrazine (Table 3). However, SFO kinetics that is the preferred option to derive regulatory degradation endpoints failed to capture the change in atrazine concentration during the incubation with the first application of atrazine (Fig. 2, Online Resources 12–18). To remedy this, we generated a model that took into account the exponential growth of atrazine degraders. This microbial growth model gave better fits to all data sets with much tighter residuals (Fig. 2, Online Resources 12–18) than existing accepted methods, which do not consider microbial growth and adaptation between applications as only one application is modelled at a time. Models that build in growth of bacteria during an adaptation phase may not only have widespread applicability in studies of adaptation to pesticides, but also other microbially catalysed processes in natural and agricultural environments, such as monitoring the numbers of degraders involved in bioremediation (Fuentes et al. 2016).

The parameters obtained from these fits indicated that a key variable between soils is the initial number of atrazine degraders prior to atrazine addition. This varied by up to three orders of magnitude between the soils (Online Resource 19), but the final rates of atrazine degradation after adaptation were remarkably stable. It is worth noting that the highest initial numbers of atrazine-degrading organisms used to fit the data were over 3000 times less than after adaptation, which may help explain why atrazine-degrading genes were initially undetectable in this study.

### The repertoire of the atrazine-degrading genes differed between the soils exhibiting accelerated degradation

The final objective of this study was to examine the impact of soil properties on atrazine degradation and its genetic potential. It was shown that all soils that exhibited accelerated degradation contained *trzN*. TrzN has been identified as being more prevalent than the alternative atrazine chlorohyrolase; AtzA (Arbeli and Fuentes 2010) attributed to its catalytic superiority and wider substrate against a range of triazine herbicides (Shapir et al. 2007; Shapir et al. 2005). In addition, *atzA* and *atzB* were detected in six of the eight soils showing accelerated degradation. AtzB enables nitrogen to be obtained from atrazine (Seffernick et al. 2007). Although AtzA is a homologue of TrzN and having both enzymes is not required to degrade atrazine, it has been suggested that communities with both may be more tolerant to new environmental conditions (Udikovic-Kolic et al. 2012). *AtzC* could only be detected in GA, which provides isopropylamine released from the *s*-triazine ring which can be used as carbon, nitrogen and/or energy sources for bacterial growth (Strong et al. 2002). In agreement with other studies, few genes of the lower atrazine-degrading pathway were detected (Udikovic-Kolic et al. 2012) possibly due to atrazine not being the primary substrate for bacteria in most soils that are not contaminated with high concentrations of atrazine or alternatively that other unidentified pathways can catalyse these metabolic reactions.

The atrazine-degrading genes have been identified to be highly conserved in pure cultures and the environment (De Souza et al. 1998a; Sagarkar et al. 2013) and were identical in this study to those identified previously (De Souza et al. 1998a; Sagarkar et al. 2013; Mulbry et al. 2002).

### The capacity for accelerated degradation in an acidic soil was restored upon neutralisation

GRS was the only soil not to exhibit accelerated degradation. Instead, atrazine accumulated after each application and the atrazine-degrading genes were not detected in this soil. This soil had the highest carbon content, which has previously been associated with reducing atrazine mineralisation by providing an alternative carbon source to atrazine and/or increasing sorption (Popov et al. 2005; Ngigi et al. 2013). Although the carbon content may have contributed to the accelerated degradation of GRS, the PCA analysis showed that a low pH was the defining feature of GRS (Fig. 5). A low pH (<6) has previously been associated with inhibiting accelerated degradation of atrazine (Houot et al. 2000; Mueller et al. 2010), and a similar observation was made by Singh et al. (2003) who saw for the insecticide chlorpyrifos that upon transfer of a pesticide degrader to an acidic soil, the identified degrader was no longer detectable. Here, we showed that the biological potential for atrazine degradation was present in low pH, non-degrading soils (GRS) and was expressed following amendment of these soils to neutral conditions, which allowed recovery of accelerated degradation, an activity that was correlated with the detection of known atrazine-degrading genes. This is the first study to demonstrate that the low pH soils retain the genetic potential for atrazine biodegradation. The vast majority of European agricultural soils have a soil pH of 5–7; therefore, the abundance of the *atz*/*trz* genes could mean a broad range of soils have the potential for accelerated degradation.

## Conclusions

The accelerated degradation of atrazine occurred in soils that had not been treated with the pesticide or its homologues, at agriculturally relevant levels, and was related to the detection of the atrazine-degrading genes. The atrazine-degrading genes were found in all of the soils tested but were shown to vary dependent on soil properties, demonstrating that the ability to degrade a pesticide is widespread and can be retained for many years. We suggest that accelerated degradation should be explicitly considered in the risk assessment process to gain a more realistic view of pesticide efficacy and fate.

## Electronic supplementary material

ESM 1 (DOCX 255 kb)

ESM 2 (XLSX 5459 kb)
