# Supplementary material for: Microbial changes linked to the accelerated degradation of the herbicide atrazine in a range of temperate soils
Source: Environ Sci Pollut Res Int. 2017 Jan 20;24(8):7359–74. doi: 10.1007/s11356-017-8377-y (PMC5383679; doi:10.1007/s11356-017-8377-y)
Supplement: Supplementary file 1 — (DOCX 255 kb) [file 11356_2017_8377_MOESM1_ESM.docx]

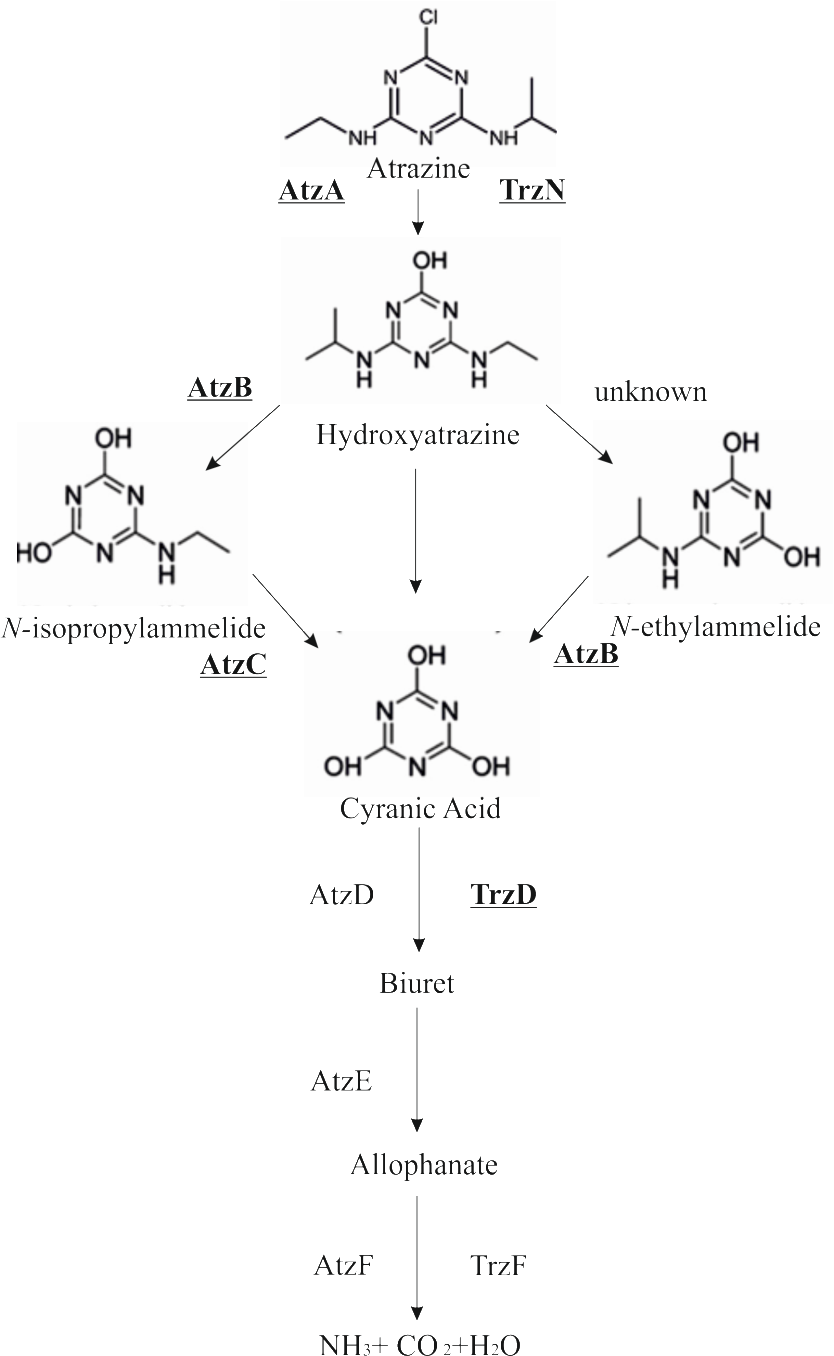


**Online Resource 1:** Microbial hydrolytic atrazine degrading pathway. The enzymes that mediate each catalytic step are shown next to the arrows. For each gene detected in this study the enzyme it encodes is in bold and underlined. Enzymes that mediate each catalytic step are: AtzA/TrzN: atrazine chlorohydrolase; AtzB: hydroxyatrazine hydrolase; AtzC: N-isopropylammelide hydrolase; AtzD/TrzD: cyanuric acid hydrolase; AtzE: biuret hydrolase and AtzF/TrzF: Allophanate hydrolase

**Online Resource 2:** Summary of the sampling regime used for the three major experiments conducted in this project. Soil; CS: Cotril set aside, CA: Cotril agricultural, MS: Mount set aside, MA: Mount agricultural, GRS: Grange set aside, GRA: Grange agricultural, GA: Ganthorpe agricultural, GS_2012: Ganthorpe set aside collected in 2012, GA_2012: Ganthorpe agricultural collected in 2012, GRA_pH: Grange agricultural collected in 2014 for pH experiment, GRS_pH: Grange set aside soil collected in 2014 for pH experiment, GRSa: GRSa_pH after neutralisation and GRAa: GRAa_pH after acidification

|  | *Microbial community changes* | *Effect of soil properties on accelerated degradation* | *Effect of pH on accelerated degradation* |
| --- | --- | --- | --- |
| Analyses conducted | ATP extraction, pyrosequencing, HPLC - atrazine detection, Q-PCR, PCR - atrazine degrading gene detection | HPLC - atrazine detection, PCR - atrazine degrading gene detection * | HPLC - atrazine detection, PCR - atrazine degrading gene detection ** |
| Soils (total number of soils) | GA_2012; GS_2012 (2) | GA_2012; GS_2012; GA; CS; CA; MS; MA; GRS; GRA (9***) | GRA_pH; GRS_pH; GRAa; GRSa (4) |
| Number of jars treated with atrazine | 12 | 4 | 4 |
| Number of controls jars (not treated with atrazine) | 4 | 4 | 4 |
| Time points subsamples removed, after each atrazine application (days) | 1^st^: 0, 1, 3, 7, 14, 28, & 60; 2^nd^: 0, 1, 3, 7, 14, 28 and 3^rd^: 0, 1, 3, 7, 14, 28 | 1^st^: 0, 1, 3, 7, 14, 28, 45 & 60; 2^nd^: 0, 1, 3, 7, 14, 28 and 3^rd^: 0, 1, 3, 7, 14, 28 | 1^st^: 0, 1, 3, 7, 14, 28, 45 & 60; 2^nd^: 0, 1, 3, 7, 14, 28 |
| Amount of soil sample removed at each time point | ~ 6 g | ~ 6 g | ~ 6 g |
| Amount of soil used for DNA extraction | ~ 5 g | ~ 5 g | ~ 5 g |
| Amount of soil used for atrazine extraction | ~1 g | ~1 g | ~1 g |

*a separate sterile experiment was conducted with a new batch of GRA to establish that atrazine was being degraded microbially

**sorption of atrazine was investigated using the soil remaining in the jars after the experiment had ceased

***Dataset consisted of 9 soils as GA_2012 and GS_2012 were utilised for this investigation

**Online Resource 3:** Pesticide history of the agricultural soils used in this study. The main active substance applied for each year and the type of pesticide is in brackets as follows; I: Insecticide, H: Herbicide, F: Fungicide. Key; I: Insecticide, H: Herbicide, F: Fungicide. Soil identifier; CA: Cotril agricultural, GA: Ganthorpe agricultural, GRA: Grange agricultural and MA: Mount agricultural.

| **Year** | **CA** | **GA** | **GRA** | **MA** |
| --- | --- | --- | --- | --- |
| **2008** | - | Quinmerac & metazachlor (H) | ***-*** |  |
| **2009** | - | α-Cypermethrin (I), Cypermethrin (I), Flufenacet & Pendimethalin (H) | Oxamyl (N/I), Linuron & Trifluralin (H), Diquat (H), Rimsulfuron (H), Fluazinam (F), Fluopiolide + propamocarb hydrochloride, MCPA (H), Cymoxanil/Cymoxanil + mancozeb (F), Cyazofamid (F), Cymoxanil + famoxadone (F), (I), Diquat (H), Metribuzin (H) | - |
| **2010** | - | Cypermethrin (I), Flufenacet & Pendimethalin (H), α-Cypermethrin (I), Epoxiconazole (F), Chlorothalonil (F), Prosulfocarb (H) | Pendimethalin (H), Diflufenican (H),Florasulam + fluroxypyr (H), Chlorothalonil (F), Pyraclostrobin (F) | Flupyrsulfuron-methyl, Metsulfuron-methyl (H), Acetamiprid (I), Chlorothalonil + picoxystrobin (F) |
| **2010 -11** | Picolinaten & Pendimethalin (H), Diflufenican (H), Cypermethrin (I) | - | - | - |
| **2011** | Boscalid & Epoxiconazole (F), Prothioconazole & Trifloxystrobin (F), Pyraclostrobin (F), Fluroxypyr & Flurasulum (H) | Fluoxastrobin (F), Fenpropimorph (F), Prosulfocarb (H) | Glyphosate (H), lambda-cyhalothrin (I), Propiconazole (F) Propaquizafop (H), cypermethrin (I), Prothioconazole (F), Tepraloxydim (H) | Abamectin (I), Cymoxanil (F), Fluroxypyr methyl heptyl ester & Florasulam (H) |
| **2011-12** | Flupyrsulfuron & tribenuron-methyl (H), Quizalofop-p-tefuryi (H), Cypermethrin (I), Prothioconazole & Tebuconazole (F), Tau fluvalinate (I) | - | - |  |
| **2012** | Glyphosate (H) | Metamitron (H), Ethofumesate & Phenmedipham (H), Lenacil (H) | Prothioconazole (fungicide), Propiconazole (F), Epoxiconazole (F), Azoxystrobin (F), lambda-cyhalothrin (I), Epoxiconazole & Fluxapyroxad (F), Bixafen & Prothioconazole & Fluoxastrobin (F), Chlorothalonil (F), Pendimethalin (H), Diflufenican (H), Cypermethrin (I), Glyphosate (H) |  |
| **2012-13** | Prothiocan & Tebuconazole (F), Bixafen & Prothioconazole & Tebuconazole (F), Boscalid & Epoxiconazole (F), Chlorathalonil | - |  |  |
| **2013** | Mesosulfuron-methyl & iodosulfuron-methyl sodium (H), Metrafenone & Epoxiconazole & Fenpropimorph (F) | Thifensulfuron methyl & metsulfuron methyl (H), Pinoxaden & Cloquintocet-mexyl (H), Prothioconazole & Bixafen (F), mecoprop-P (H) | Bixafen & Prothioconazole (F), Metsulfuron-methyl & tribenuron-methyl (H), Florasulam & fluxypyr (H), Fluoastrobin & prothioconazole + trifloxystrobin (F), manganese, Glyphosate (H) | Lambda-cyhalothrin (I), Prosulfuron (H), Picolinafen & Pendimethalin (H), Phenmedipham (H),Triflusulfuron-methyl (H) |
| **2014** | - | - | Bixofen & Profalconanazole (F), Chloromoquat, Trinexapac-ethyl, Manganese | - |

**Online Resource 4:** Soil pH throughout the duration of atrazine dissipation, in GRAa and GRSa soils that had had their pH amended. Grange set aside soil (GRS) had a pH 5.4 and had 2 mg g^-1^ of lime (Ca(OH)_2_) added to maintain ~ pH 7, and was referred to as GRSa (GRS amended). The Grange agricultural soil (GRA) soil had a pH 6.2, had 8 mg g^-1^ of aluminium sulphate (Al_2_(SO_4_)_3_) added and was maintained at ~ pH 4, and referred to as GRAa (GRA amended). Soil pH was monitored in a non-atrazine treated control pot weekly for each soil and each amendment added as required, followed by moisture adjustments

**Online Resource 5:** Variation in the ATP content between three soil sub-samples from the same larger soil sample. Error bars indicate the standard error in relative light units between duplicate aliquots of each soil sub-sample (n = 2).

**Online Resource 6:** The efficiency of the primer pair *16S rRNA* gene was assessed by plotting the cycle threshold value (*C_t_*) at each concentration against the logarithm of the ten-fold dilutions of the sample. The slope of a linear-regression trendline is indicative of primer efficiency.

**Online Resource 7:** The efficiency of the primer pair *trzN* gene was assessed by plotting the cycle threshold value (*C_t_*) at each concentration against the logarithm of the ten-fold dilutions of the sample. The slope of a linear-regression trendline is indicative of primer efficiency.

**Online Resource 8:** Barcodes used in pyrosequencing of the Ganthorpe set aside (GS_2012) and agricultural (GA_2012) soils collected in 2012. The sample associated with each barcode is as follows; soil history; set aside (S) or agricultural (A), duration in days under incubation conditions; 0 days (0) or 120 days (120) atrazine treatment; treated (T) or control (C), followed by an individual replicate number.

| Sample Identity | Barcode sequence |
| --- | --- |
| S0_c1 | TACTCTCGTG |
| S0_c2 | TAGAGACGAG |
| S0_c4 | ACATACGCGT |
| A0_c1 | AGCGTCGTCT |
| A0_c2 | AGTACGCTAT |
| A0_c3 | ATAGAGTACT |
| S120_T1 | AGACGCACTC |
| S120_T2 | AGCACTGTAG |
| S120_c3 | CGTGTCTCTA |
| S120_c4 | CTCGCGTGTC |
| A120_T1 | CATAGTAGTG |
| A120_T2 | CGAGAGATAC |
| A120_c3 | CGTCTAGTAC |
| A120_c4 | TCTACGTAGC |

**Online Resource 9:** Number of partial *16S RNA* gene sequences remaining in each soil sample after quality filtering in QIIME. Post-QIIME processing follows the removal of sequences with 4 or more primer mismatches and/ a minimum average quantity score less than 30.

| **Sample Id.** | **Pre-processing** | **Post-QIIME processing** |
| --- | --- | --- |
| S0_c1 | 8578 | 7428 |
| S0_c2 | 9748 | 8918 |
| S0_c4 | 8736 | 7606 |
| A0_c1 | 8545 | 7056 |
| A0_c2 | 11584 | 9551 |
| A0_c3 | 12052 | 11304 |
| S120_T1 | 4516 | 3459 |
| S120_T2 | 7183 | 6346 |
| S120_c3 | 6169 | 5136 |
| S120_c4 | 6242 | 5277 |
| A120_T1 | 7903 | 7109 |
| A120_T2 | 3461 | 2910 |
| A120_c3 | 7336 | 6888 |
| A120_c4 | 7829 | 7359 |

**Online Resource 10:** Parameters used to model atrazine dissipation using Single First Order according to FOCUS (2006). M0 is the amount of the atrazine on day 0 (% of applied) and k is the rate constant. Soil; CS: Cotril set aside, CA: Cotril agricultural, MS: Mount set aside, MA: Mount agricultural, GRS: Grange set aside, GRA: Grange agricultural, GA: Ganthorpe agricultural, GS_2012: Ganthorpe set aside collected in 2012, GA_2012: Ganthorpe agricultural collected in 2012, GRA_pH: Grange agricultural collected in 2014 for pH experiment, GRS_pH: Grange set aside soil collected in 2014 for pH experiment, GRSa: GRSa_pH after neutralisation and GRAa: GRAa_pH after acidification.

|  | |  | | ***First application*** | | |  | | ***Second application*** | | |  | | ***Third application*** | |
| --- | --- | --- | --- | --- | --- | --- | --- | --- | --- | --- | --- | --- | --- | --- | --- |
| ***Soil*** |  | | ***M0* (%)** | | ***k*** |  | | ***M0* (%)** | | ***k*** |  | | ***M0* (%)** | | ***k*** |
| CS |  | | 74.87 | | 0.02 |  | | 92.81 | | 0.24 |  | | 80.33 | | 0.71 |
| CA |  | | 76.65 | | 0.02 |  | | 96.38 | | 0.67 |  | | 74.46 | | 0.54 |
| MS |  | | 71.27 | | 0.04 |  | | 80.77 | | 0.44 |  | | 77.32 | | 0.76 |
| MA |  | | 72.27 | | 0.04 |  | | 93.63 | | 0.25 |  | | 72.72 | | 0.71 |
| GRS |  | | 76.35 | | 0.01 |  | | 109.6 | | 0.01 |  | | 146.1 | | 0.01 |
| GRA |  | | 68.23 | | 0.03 |  | | 87.61 | | 0.5 |  | | 68.25 | | 0.53 |
| GA |  | | 74.04 | | 0.019 |  | | 89.9 | | 0.61 |  | | 81.39 | | 0.37 |
| GS_2012 |  | | 74.64 | | 0.032 |  | | 64.24 | | 0.39 |  | | 62.58 | | 0.45 |
| GA_2012 |  | | 81.37 | | 0.034 |  | | 63.76 | | 0.64 |  | | 62.97 | | 0.69 |
| GRS_pH |  | | 63.06 | | 0.02 |  | | 101.9 | | 0.02 |  | | - | | - |
| GRSa |  | | 70.17 | | 0.04 |  | | 76.44 | | 0.66 |  | | - | | - |
| GRA_pH |  | | 57.93 | | 0.03 |  | | 83.19 | | 0.45 |  | | - | | - |
| GRAa |  | | 59.21 | | 0.02 |  | | 96.63 | | 0.03 |  | | - | | - |

**Online Resource 11:** Parameters used to model the dissipation of atrazine in non-sterile and sterile soils originating from the Grange agricultural (GRA) soil. M0 is the amount of the atrazine on day 0 and k is the rate constant. DT_50_ is the time taken for 50 % of atrazine to dissipate and Χ^2^ measures the deviation of the measured data from the SFO model.

| ***Soil*** | ***M0* (%)** | ***k*** | ***Χ^2^*** | ***DT_50_* (days)** |
| --- | --- | --- | --- | --- |
| GRA (non_sterile) | 64.71 | 0.034 | 3.28 | 20.35 |
| GRA (sterile) | 54.63 | 0.0064 | 4.98 | 107.72 |

**Online Resource 12:** Comparison of modelling approaches for the dissipation of atrazine in Cotril agricultural soil (CA) over three applications. Using the regulatory single first order (SFO) approach with each application modelled separately (bottom) and the ‘growth linked model’ described in this study (top). The ‘growth linked model’ enabled all applications to be modelled simultaneously. In both modelling approaches the model fit of % atrazine remaining is shown as a solid black line and individual soil sub-samples as diamonds (n = 4). For the ‘growth linked model’ the grey dashed line represents the number of atrazine degraders.

**Online Resource 13:** Comparison of modelling approaches for the dissipation of atrazine in Cotril set aside soil (CS) over three applications. Using the regulatory single first order (SFO) approach with each application modelled separately (bottom) and the ‘growth linked model’ described in this study (top). The ‘growth linked model’ enabled all applications to be modelled simultaneously. In both modelling approaches the model fit of % atrazine remaining is shown as a solid black line and individual soil sub-samples as diamonds (n = 4). For the ‘growth linked model’ the grey dashed line represents the number of atrazine degraders.

**Online Resource 14:** Comparison of modelling approaches for the dissipation of atrazine in Ganthorpe agricultural soil (GA) over three applications. Using the regulatory single first order (SFO) approach with each application modelled separately (bottom) and the ‘growth linked model’ described in this study (top). The ‘growth linked model’ enabled all applications to be modelled simultaneously. In both modelling approaches the model fit of % atrazine remaining is shown as a solid black line and individual soil sub-samples as diamonds (n = 4). For the ‘growth linked model’ the grey dashed line represents the number of atrazine degraders.

**Online Resource 15:** Comparison of modelling approaches for the dissipation of atrazine in Mount agricultural soil (MA) over three applications. Using the regulatory single first order (SFO) approach with each application modelled separately (bottom) and the ‘growth linked model’ described in this study (top). The ‘growth linked model’ enabled all applications to be modelled simultaneously. In both modelling approaches the model fit of % atrazine remaining is shown as a solid black line and individual soil sub-samples as diamonds (n = 4). For the ‘growth linked model’ the grey dashed line represents the number of atrazine degraders..

**Online Resource 16:** Comparison of modelling approaches for the dissipation of atrazine in Mount set aside soil (MS) over three applications. Using the regulatory single first order (SFO) approach with each application modelled separately (bottom) and the ‘growth linked model’ described in this study (top). The ‘growth linked model’ enabled all applications to be modelled simultaneously. In both modelling approaches the model fit of % atrazine remaining is shown as a solid black line and individual soil sub-samples as diamonds (n = 4). For the ‘growth linked model’ the grey dashed line represents the number of atrazine degraders.

**Online Resource 17:** Comparison of modelling approaches for the dissipation of atrazine in Ganthorpe agricultural soil collected in 2012 (GA_2012) over three applications. Using the regulatory single first order (SFO) approach with each application modelled separately (bottom) and the ‘growth linked model’ described in this study (top). The ‘growth linked model’ enabled all applications to be modelled simultaneously. In both modelling approaches the model fit of % atrazine remaining is shown as a solid black line and individual soil sub-samples as diamonds (n = 4). For the ‘growth linked model’ the grey dashed line represents the number of atrazine degraders.

**Online Resource 18:**  Comparison of modelling approaches for the dissipation of atrazine in Ganthorpe set aside soil collected in 2012 (GS_2012) over three applications. Using the regulatory single first order (SFO) approach with each application modelled separately (bottom) and the ‘growth linked model’ described in this study (top). The ‘growth linked model’ enabled all applications to be modelled simultaneously. In both modelling approaches the model fit of % atrazine remaining is shown as a solid black line and individual soil sub-samples as diamonds (n = 4). For the ‘growth linked model’ the grey dashed line represents the number of atrazine degraders.

**Online Resource 19:** Variation in parameters used in the ‘growth linked model’ between the 8 soils that demonstrated AD. Parameters; atr; starting amount of atrazine; Y: % of atrazine that is available for degradation; k1: exponential atrazine decay rate; n: length of the timestep (typically set at 0.01 days) used in the modelling; No: size of the atrazine degrader population at day 0, V: maximum rate of atrazine removal and Ks: Michaelis constant representing the concentration of atrazine that gives half the maximum rate of hyperbolic atrazine degradation. Soil identifier; GA_2012: Ganthorpe agricultural collected in 2012, GS_2012: Ganthorpe set aside collected in 2012, CS: Cotril set aside, CA: Cotril agricultural, MA: Mount agricultural, MS: Mount set aside, GRA: Grange agricultural, GRS: Grange set aside and GA: Ganthorpe agricultural.

**Online Resource 20:** Closest relatives of the atrazine degrading genes sequenced. Gene identities were obtained from the NCBI. Enzymes that genes encode; *atzA/trzN*: atrazine chlorohydrolase; *atzB*: hydroxyatrazine hydrolase; *atzC*: N-isopropylammelide hydrolase and *trzD*: cyanuric acid hydrolase.

| ***Gene*** | ***Accession.version*** *(NCBI, blastn)* | ***Organism*** | ***% identity*** |
| --- | --- | --- | --- |
| *atzA* | HQ400756.1 | *Aminobacter aminovorans* strain Sal 1-3 atrazine chlorohydrolase gene, partial cds | 100 |
| *atzB* | KF453508.1 | *Arthrobacter* sp. DNS10 hydroxyatrazine hydrolase (atzB) gene, complete cds. | 100 |
| *atzC* | EF088654.1 | *Nocardioides* sp. CMU5 AtzC (atzC) gene, partial cds | 100 |
| *trzN* | KF453507.1 | *Arthrobacter* sp. DNS10 triazine hydrolase (trzN) gene, complete cds. | 100 |
| *trzD* | HE716865.1 | *Pseudomonas* sp. AK_CAN1 partial trzD gene for cyanuric acid amidohydrolase, isolate AK_CAN1. | 100 |

**Online Resource 21:** ATP content of soils over three applications of atrazine measured in average relative light units detected 7 days after the first (1), second (2) and third (3) applications of atrazine. A: agricultural soil (GA_2012) & S: set aside soil (GS_2012). Error bars indicate the standard error in RLUs between soils application 1 (n = 4), application 2 (n = 3) and application 3 (n = 4).

Online Resource 22: Eigen values for five principal components, based on the variation between nine temperate soils based on their properties.

| PC | Eigenvalues | Variation (%) | Cumulative variation (%) |
| --- | --- | --- | --- |
| 1 | 6.12 | 68 | 68 |
| 2 | 1.8 | 20 | 87.9 |
| 3 | 0.753 | 8.4 | 96.3 |
| 4 | 0.185 | 2.1 | 98.3 |
| 5 | 0.111 | 1.2 | 99.6 |

Online Resource 23: Eigenvectors for Coefficients in the linear combinations of variables making up the first 5 principal components, based on the soil properties of nine temperate soils. Key; PC: principal component; MC: moisture content, MWHC: Maximum water holding capacity, N: nitrogen, C: carbon, C:N; carbon to nitrogen ratio.

| Variable | PC1 | PC2 | PC3 | PC4 | PC5 |
| --- | --- | --- | --- | --- | --- |
| pH In H_2_O | -0.156 | 0.636 | 0.333 | 0.382 | 0.185 |
| Sand (%) | 0.387 | -0.147 | -0.227 | 0.175 | 0.078 |
| Silt (%) | -0.371 | 0.012 | 0.388 | -0.06 | -0.635 |
| Clay (%) | -0.359 | 0.288 | 0.012 | -0.289 | 0.586 |
| Total N (%) | -0.375 | 0.121 | -0.368 | -0.115 | -0.275 |
| Organic C (%) | -0.391 | -0.146 | -0.147 | -0.234 | -0.013 |
| C:N ratio | -0.139 | -0.578 | 0.596 | -0.109 | 0.303 |
| Mean MC (%) | -0.348 | -0.266 | -0.087 | 0.81 | 0.055 |
| Mean MWHC (%) | -0.355 | -0.224 | -0.408 | 0.005 | 0.205 |

**Online Resource 24:** Principal component scores for nine temperate soils. Soil; CA: Cotril agricultural, CS: Cotril set aside, MA: Mount agricultural, MS: Mount set aside, GRA: Grange agricultural, GRS: Grange set aside, GA: Ganthorpe agricultural, GS_2012: Ganthorpe set aside collected in 2012 and GA_2012: Ganthorpe agricultural collected in 2012.

| Soil | SCORE1 | SCORE2 | SCORE3 | SCORE4 | SCORE5 |
| --- | --- | --- | --- | --- | --- |
| CA | 2.27 | -0.02 | 0.08 | 0.49 | 0.37 |
| CS | 0.16 | -0.27 | -1.38 | 0.49 | -0.17 |
| MA | -4.01 | 0.58 | 0.06 | -0.29 | 0.47 |
| MS | -2.56 | 2.41 | 0.19 | 0.23 | -0.31 |
| GRA | -0.30 | -1.28 | 1.77 | 0.00 | -0.26 |
| GRS | -2.36 | -2.46 | -0.65 | -0.10 | -0.08 |
| GA | 2.22 | 0.14 | 0.52 | 0.26 | 0.33 |
| GS_2012 | 2.47 | 0.50 | -0.46 | -0.85 | 0.10 |
| GA_2012 | 2.11 | 0.40 | -0.13 | -0.23 | -0.45 |

**Online Resource 25:** Sorption of atrazine as a function of atrazine concentrations in soils of different pH. The Grange set aside amended soil (GRSa) and Grange agricultural soil (GRA_pH) had near neutral pH values of ~ pH 7 and pH 6.2, while the Grange set aside soil (GRS_pH) and Grange agricultural amended soil (GRAa) had acidic pHs of 5.4 and ~ pH 4, respectively. Atrazine was added to sub-samples of each soil at a range of concentrations and sorption calculated. Symbols are duplicate samples and lines are the Freundlich isotherms based on the kf (sorption coefficient) and 1/n values (Freundlich exponent). The following values were used to fit the isotherms; kf: GRS: 3.86, GRSa: 3.26, GRA: 3.12 and GRAa: 3.55 and 1/n: GRS: 0.74, GRSa: 0.77, GRA: 0.69 and GRAa: 0.72. *shows that the pH of the amended soils (GRSa and GRAa) are only approximate as there was minor variation in their soil pH throughout the experiment (Online Resource 4).

*****


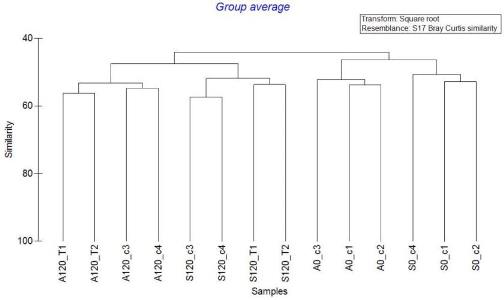


**Online Resource 26:** Bacterial community comparison for between the Ganthorpe agricultural (GA_2012) and set aside (GS_2012) soils collected in 2012, examined using group-average clustering based on the data from Bray-Curtis similarity matrices. Labels based on soil history; set aside (S) or agricultural (A), time sample was removed; at the beginning of the study (0) or 120 days after atrazine addition (120) and whether the bacterial communities originated from soil that had been treated with atrazine (T) or whether the bacterial community originated from control soil (C), followed by an individual replicate number
